# The Long-Term Culture of Human Fibroblasts Reveals a Spectroscopic Signature of Senescence

**DOI:** 10.3390/ijms23105830

**Published:** 2022-05-23

**Authors:** Sandra Magalhães, Idália Almeida, Cátia D. Pereira, Sandra Rebelo, Brian J. Goodfellow, Alexandra Nunes

**Affiliations:** 1 iBiMED—Institute of Biomedicine, Department of Medical Sciences, University of Aveiro, Agra do Crasto, 3810-193 Aveiro, Portugal; sandra.vicencia@ua.pt (S.M.); idalia24@ua.pt (I.A.); daniela.pereira@ua.pt (C.D.P.); srebelo@ua.pt (S.R.); 2 CICECO—Aveiro Institute of Materials, Department of Chemistry, University of Aveiro, Campus Universitário de Santiago, 3810-193 Aveiro, Portugal; brian.goodfellow@ua.pt

**Keywords:** aging, cell senescence, human fibroblasts, FTIR spectroscopy, NMR spectroscopy

## Abstract

Aging is a complex process which leads to progressive loss of fitness/capability/ability, increasing susceptibility to disease and, ultimately, death. Regardless of the organism, there are some features common to aging, namely, the loss of proteostasis and cell senescence. Mammalian cell lines have been used as models to study the aging process, in particular, cell senescence. Thus, the aim of this study was to characterize the senescence-associated metabolic profile of a long-term culture of human fibroblasts using Fourier Transform Infrared and Nuclear Magnetic Resonance spectroscopy. We sub-cultivated fibroblasts from a newborn donor from passage 4 to passage 17 and the results showed deep changes in the spectroscopic profile of cells over time. Late passage cells were characterized by a decrease in the length of fatty acid chains, triglycerides and cholesterol and an increase in lipid unsaturation. We also found an increase in the content of intermolecular β-sheets, possibly indicating an increase in protein aggregation levels in cells of later passages. Metabolic profiling by NMR showed increased levels of extracellular lactate, phosphocholine and glycine in cells at later passages. This study suggests that spectroscopy approaches can be successfully used to study changes concomitant with cell senescence and validate the use of human fibroblasts as a model to monitor the aging process.

## 1. Introduction

Aging is a complex process that occurs in almost all organisms and, even though each organism ages differently, there are common features, which allows the process to be studied in a wide range of models. In 2016, the loss of proteostasis and cell senescence was described as hallmarks of aging [1]. The integrity of cellular proteome is maintained by the machinery of the proteostasis network. This is a dynamic system and ensures a proper balance between the synthesis, folding and degradation of proteins and inhibits the accumulation of misfolded and/or toxic aggregated proteins in cells under stress [2]. The loss of proteostasis is well-described in age-related diseases, with increasing levels of toxic protein aggregates being involved in diseases such as Alzheimer’s, Huntington’s and Parkinson’s diseases, or in amyotrophic lateral sclerosis [3]. Studies have shown that proteostasis is altered during aging and modeling some components of this network can provide insights into the lifespan of some organisms [4]. Cellular senescence is considered a protective mechanism for cells to avoid excessive proliferation and was first described in the 1960s in human fibroblasts [5]. This is called replicative senescence and, similar to the loss of proteostasis, senescent cells are found to accumulate during aging and age-related diseases [6].

Due to the complexity of studying aging in humans, a number of model organisms have been used to unravel molecular mechanisms associated with aging: *Drosophila melanogaster* [7] and other insects [8], *Caenorhabditis elegans* [9], *Mus musculus* [10] and also mammalian cell lines [11].

Mammalian cell lines have been used for many decades to study some features of aging such as cellular senescence [12]. Primary cells, such as skin fibroblasts, should be used, rather than immortalized cell lines. Immortalized cells can escape the control of the cell cycle and, therefore, may not acquire a senescent phenotype [1,13,14]. Another important point for the study of cellular senescence is the culture conditions. For instance, cultured cells may be exposed to fluorescent light that may cause DNA damage and the trypsinization process, commonly used as a standard procedure to sub-culture cells, and can induce cellular stress [14]. Despite these concerns, studying cellular senescence in vitro presents several advantages versus other models: it is possible to easily perform manipulations and evaluate cellular response to treatments; a high number of replicates can be produced to ensure reproducible results and [11].

Two different approaches can be used to study the mechanisms of aging using cell cultures, both having *pros* and *cons*: the first is to use a cell line derived from a young donor and sub-culture cells in vitro until they reach senescence; the other option is to culture cells from several donors with different ages and compare molecular and/or morphological markers [15]. One of the major disadvantages of the first approach is the time needed to sub-culture the same cells to senescence, which can take months. However, for accuracy, it is considered the most advantageous method to investigate aging in cell lines, since this way, we do not need to consider the heterogenicity we would have to if we were analyzing cells from different donors with different ages [14].

In order to assess cellular senescence both in vitro and in vivo, we may determine the loss of division potential, the increase in cell size and the increase in the number of multinucleated cells [14]. We may also consider evaluating changes in the metabolome of cells and relate these back to the aging process, since aging is accompanied by changes in the endo- and exocellular metabolome which can provide valuable insights into the molecular mechanism and signaling pathways altered during aging. Metabolomics studies based on cell cultures have increased over the last few years, mainly using NMR and MS-based approaches [16]. A metabolomics approach applied to cell cultures has been used to provide valuable information on cell phenotype as well as metabolic networks and to discover biomarkers with the results complementing those from human or animal models. Some drawbacks include the influence of experimental procedures of cell extraction and quenching on the metabolic profile of cells [16]. This way, Fourier Transform Infrared (FTIR) spectroscopy appears as a promising approach to study cellular metabolome, since it is possible to analyze whole cells without the need for metabolites’ extraction, which translates into less cellular stress, keeping metabolic profiles unchangeable. FTIR spectroscopy of fixed cells has already been successfully used to evaluate senescence in human fibroblasts [17].

In this study, we hypothesized that FTIR spectroscopy can be used to evaluate protein conformational changes during the process of cellular senescence. This way, we aimed to characterize the senescence-associated profile of human fibroblasts to provide insights into the aging process. To accomplish this, we performed a long-term culture of human dermal fibroblasts from a newborn donor and, using FTIR spectroscopy and NMR spectroscopy, we evaluated changes in whole cells and in the cellular exo- and endometabolome over time.

## 2. Results

### 2.1. Lamin B1 Levels in Fibroblast Nuclei Drop from Passage 5 to Passage 17

**Figure 1:**
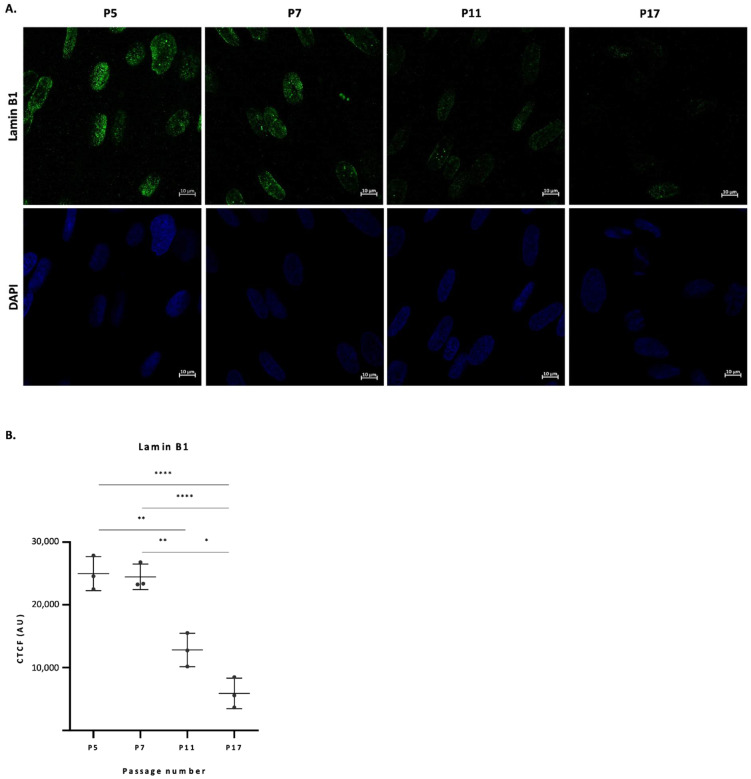
**Lamin B1 levels in fibroblast nuclei drop from passage 5 to passage 17**. (**A**) Representative image of fibroblasts’ nuclei at passages 5, 7, 11 and 17 stained with anti-lamin B1 antibody. Upper panel—antibody staining; Lower panel—DAPI staining. Images represent Z-stacks. (**B**) Quantification of lamin B1’s fluorescence levels via immunofluorescence: histogram of nuclear intensity. Data from histogram is presented as of the mean of lamin B1 intensity from 30 cells for each of the three experiments for the four analyzed passages. Scale: 10 µm. * *p* <0.05; ** *p* < 0.01; **** *p* < 0.0001. CTCF: Corrected total cell fluorescence.

Down-regulation of lamin B1 is a commonly used marker for senescence both in vitro and in vivo [18]. As fibroblasts reach passage 17, a significant decrease in cell proliferation and consequently time to confluency is observed. In fact, we were not able to sub-culture our fibroblasts beyond passage 18. To confirm that these cells have reached senescence we performed an immunocytochemical analysis at four different random timepoints (passages 5, 7, 11 and 17) using a specific anti-lamin B1 antibody. Figure 1 shows the significant decline in the Corrected Total Cell Fluorescence (CTCF) levels of lamin B1 in fibroblast nuclei for cells at higher passage numbers, compared to passages 5 and 7. Panel A shows representative images of lamin B1 and DAPI staining, and panel B shows quantitative results of lamin B1 fluorescence intensity. It can be seen that cells at passages 5 and 7 present similar levels of lamin B1. However, from passage 7 to 11, there is a pronounced decrease in the CTCF levels of this marker, which becomes more pronounced at passage 17. The decrease in the levels of lamin B1 is a characteristic of senescent cells [18], showing that these cells may have reach a status of replicative senescence.

### 2.2. FTIR Spectra Overview

**Figure 2:**
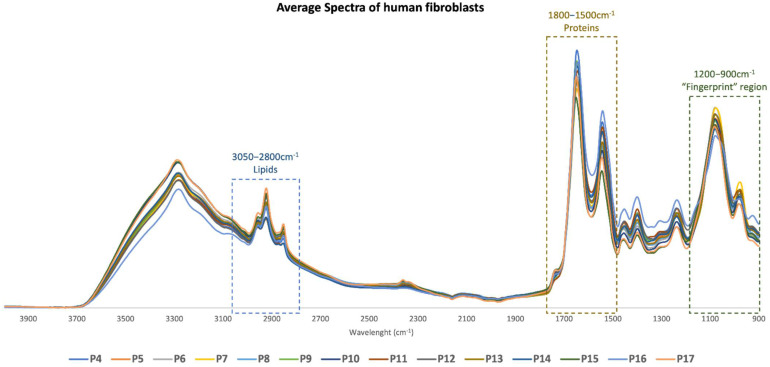
**Average FTIR spectra of human fibroblasts in the mid-infrared region from passages 4 to 17**. Average FTIR spectra of AG22153 cell line (Coriell Institute) in the mid-infrared region (4000–900 cm^−1^) in 14 different passages, from passage 4 to passage 17. Boxes represent the main spectral regions used for statistical analysis.

To elucidate the spectroscopic profile of human fibroblasts from initial passages to senescence, we acquired FTIR spectra of cells over 14 successive passages (4 to 17). The average baseline-corrected, area normalized spectra of the cells are presented in Figure 2. Spectra were area normalized to ensure that slight differences in the amount of sample placed on the Attenuated Total Reflectance (ATR) crystal would differentiate between samples.

Visual analysis of the spectra did not reveal any obvious differences between spectra from different passages. Therefore, to extract valuable information from spectral data, we performed a Partial Least Squares Regression (PLS-R) analysis after outlier removal, baseline correction and area normalization for three sub-spectral regions: 3050–2800 cm^−1^, 1800–1500 cm^−1^ and 1200–900 cm^−1^, as explained in Section 4.4.2. This analysis allowed us to unravel spectroscopic changes occurring in cells during the process of senescence and to construct a prediction model for each spectral region. For each PLS-R analysis, we chose the best factor to maximize the explained variance between samples and avoid overfitting of the model (factor 3 for the 3050–2800 cm^−1^, 1800–1500 cm^−1^ and 1200–900 cm^−1^ regions and factor 4 for the 1700–1600 cm^−1^ region). We also performed an analysis of specific peak intensities to detect changes for specific functional groups.

### 2.3. Intensity of FTIR Spectral Bands

**Figure 3:**
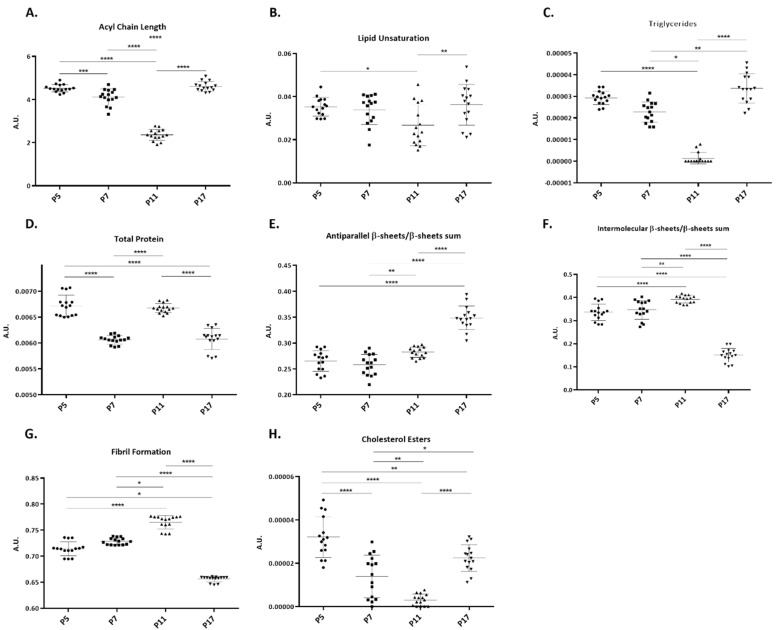
**Analysis of peak intensities of human fibroblasts from a newborn at passages 5, 7, 11 and 17.** (**A**) Acyl chain length, calculated using CH_2_ and CH_3_ peak intensities (ratio I_2851+2922_/I_2959+2871_); (**B**) Lipid unsaturation levels, calculated using the ratio between olefinic band and CH_2_ bands (ratio I_3013_/ I_2851+2922_); (**C**) Triglycerides levels, calculated using the intensity of C=O band at 1741 cm^−1^; (**D**) Total protein levels, calculated by the sum of Amide I and Amide II peaks (I_Amide II_/I_Amide I_); (**E**) Ratio antiparallel β-sheet/β-sheets’ sum, calculated using I_1693_/I_1693+1682+1628_; (**F**) Ratio intermolecular β-sheets/β-sheets’ sum, calculated using I_1628_/I_1693+1682+1628_; (**G**) Fibril formation, calculated using Amide I and Amide II peak intensities (ratio I_Amide II_/I_Amide I_)—using baseline-corrected, area-normalized and non-derived spectra and (**H**) Cholesterol esters levels, calculated using the intensity of peak at 1169 cm^−1^. Data are expressed as mean ±SD. * *p* < 0.05; ** *p* < 0.01; *** *p* < 0.001; **** *p* < 0.0001.

The intensity changes observed in some bands during cell passages were also analyzed. We used both 2nd derivative spectra and non-derivative normalized spectra from fibroblasts at passages 5, 7, 11 and 17. The results from this analysis are presented in Figure 3. All peaks that could be assigned to lipid functional groups (Figure 3A–C) presented the same profile: a progressive decrease from passages 5 to 11, followed by a significant increase up to passage 17. In the case of the peak assigned to cholesterol esters, there is also a progressive decrease in the intensity of this peak from passages 5 to 11, followed by an increase to passage 17, but to lower levels than those at passage 5 (Figure 3H).

Using the sum of amide I and amide II peaks, a decrease in protein levels from passages 5 to 7, 5 to 17 and 11 to 17 were observed. However, from passages 7 to 11, there is a significant increase (Figure 3D). For antiparallel β-sheets a progressive increase in the levels of these structures is seen, as shown in Figure 3E, which may represent an increase in oligomeric structures in cellular proteins. Intermolecular β-sheets are aggregation-prone, so the levels of these structures may reflect the levels of protein aggregation in the cell. Hence, it is not surprising that the profile for intermolecular β-sheets and fibril formation ratio is the same (panels F and G of Figure 3): a gradual increase from cells at passage 5 to cells at passage 11, followed by a pronounced and significant decrease to passage 17.

### 2.4. PLS-R Multivariate Analysis

A PLS regression model was constructed for all three sub-spectral regions to understand how the overall spectroscopic profile can be an indicator of the process of cell senescence in vitro. In fact, looking to scores plot of the PLS analysis in all three spectral regions (Supplementary Figure S1A–C), we noticed that cells at later passages (especially passages 15, 16 and 17) present a spectroscopic profile distinct to younger cells, showing that FTIR spectroscopy may be able to detect changes concomitant with senescence.

### 2.4.1. 3050–2800 cm^−1^ Region

**Figure 4:**
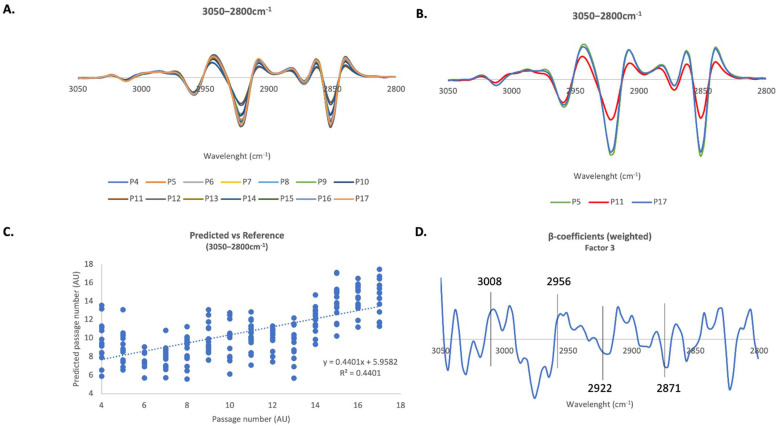
**FTIR PLS multivariate analysis of human fibroblasts in the 3050–2800 cm^−1^ region**. (**A**) Average 2nd derivative spectra of human fibroblasts in the 3050–2800 cm^−1^ region. (**B**). Average 2nd derivative spectra of fibroblasts in the 3050–2800 cm^−1^ region but only for passages P5, P11 and P17. (**C**) PLS-R Predicted vs. Reference plot of factor 3 of the second-derivative FTIR spectra of human fibroblasts in the 3050–2800 cm^−1^ region. (**D**) Respective β-coefficients plots of factor 3.

The 2nd derivative spectra of the 3050–2800cm ^−1^ region, where it is possible to observe vibrations of lipids’ functional groups, is amplified in Figure 4A. Figure 4B shows 2nd derivative spectra of only cells at passages 5, 11 and 17, and looking to the spectra, we notice that the bigger differences in spectroscopic profile in this region are verified for cells at passage 11. A PLS-R calibration model was constructed in this region using three factors and a linear correlation between spectra profile and passage number with a correlation coefficient R = 0.66 (Figure 4C) was found. An internal cross-validation performed on the model resulted in a linear correlation with an R value of 0.50.

The β-coefficient plot revealed that peaks at 3008 cm^−1^, assigned to CH groups of lipids, and 2956 cm^−1^, assigned to asymmetric vibrations of CH_3_ groups of lipids, are associated with cells at later passages. On the other hand, peaks at 2922 cm^−1^ and 2871 cm^−1^, associated with asymmetric vibrations of CH_2_ groups of lipids and with symmetric vibrations of CH_3_ groups of lipids, respectively, are associated with cells at earlier passages (Figure 4D).

### 2.4.2. 1800–1500 cm^−1^ Region

**Figure 5:**
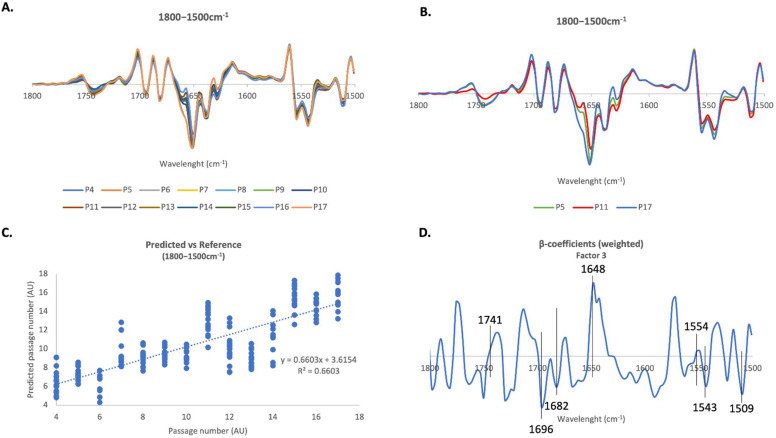
**FTIR PLS multivariate analysis of human fibroblasts in the 1800–1500 cm^−1^ region**. (**A**) Average 2nd derivative spectra of human fibroblasts in the 1800–1500 cm^−1^ region. (**B**) Average 2nd derivative spectra of fibroblasts in the 3050–2800 cm^−1^ region but only for passages P5, P11 and P17. (**C**) PLS-R Predicted vs. Reference plot of factor 3 of the second-derivative FTIR spectra of human fibroblasts in the 1800–1500 cm^−1^ region. (**D**) Respective β-coefficients plots of factor 3.

The 1800–1500 cm^−1^ region has two major peaks which specifically related to the proteins present in the analyzed samples: the amide I and amide II peaks. The amide I peak is sensitive to changes in protein secondary structure, so any changes related to changes in cellular proteostasis that may occur in cells during senescence may be observed here. The amplified 2nd derivative spectra from fibroblasts in this region can be seen in Figure 5A. Again, Figure 5B shows 2nd derivative spectra of only cells at passages 5, 11 and 17, and looking to the spectra, we notice that the bigger differences in spectroscopic profile in this region are verified for cells at passage 17, comparing to passages 5 and 11. PLS-R multivariate analysis indicated a strong positive linear correlation between spectra profile and passage number (correlation coefficient R = 0.81) (Figure 5C). Internal cross-validation retrieved a correlation coefficient of 0.78.

Looking at Figure 5D, the β-coefficients associated with this correlation show higher levels of C=O structures (peak at 1741 cm^−1^) at higher passages. In addition, younger cells seem to be characterized by β-sheet (peak at 1682 cm^−1^) and antiparallel β-sheet (peak at 1696 cm^−1^) structures, whereas cells at later passages are characterized by helical structures, as revealed by the major peak at 1648 cm^−1^. This model did not reveal any important information from the amide II sub-peaks (1554 cm^−1^ to 1509 cm^−1^), as one of the sub-peaks is associated with cells at higher passages (1554 cm^−1^) and two associated with cells at initial passages (1543 cm^−1^ and 1509 cm^−1^).

#### 2.4.3. 1200–900 cm^−1^ Region

**Figure 6:**
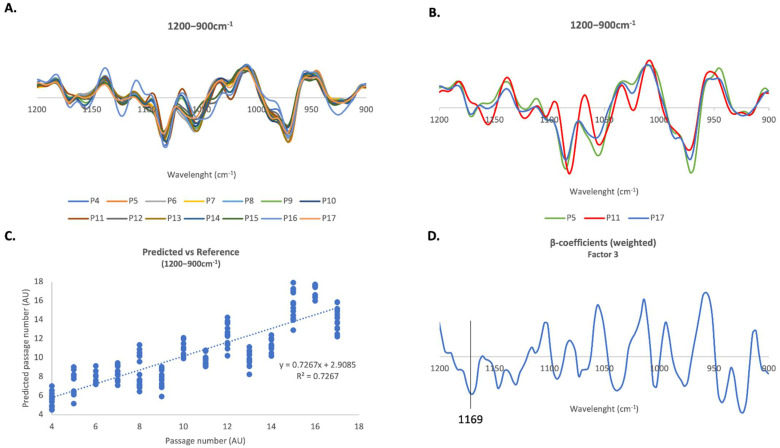
**FTIR PLS multivariate analysis of human fibroblasts in the 1200–900 cm^−1^ region**. (**A**) Average 2nd derivative spectra of human fibroblasts in the 1200–900 cm^−1^ region. (**B**) Average 2nd derivative spectra of fibroblasts in the 3050–2800 cm^−1^ region but only for passages P5, P11 and P17. (**C**) PLS-R Predicted vs Reference plot of factor 3 of the second-derivative FTIR spectra of human fibroblasts in the 1200–900 cm^−1^ region. (**D**) Respective β-coefficients plots of factor 3.

The last spectral region analyzed using PLS-R was the so-called *fingerprint region*, between 1200 and 900 cm^−1^. This region has vibrations from functional groups of biomolecules such as, nuclei acids, carbohydrates and lipids such as cholesterol. The 2nd derivative spectra used for the PLS-R analysis are plotted in Figure 6A. Figure 6B clearly shows that cells at passages 5, 11 and 17 present distinct spectroscopic profiles. The results from PLS analysis in all cell passages revealed a strong positive linear correlation between spectral data and passage number (correlation coefficient R = 0.85, internal cross-validation R = 0.83) (Figure 6C).

Looking at Figure 6D, the β-coefficients indicate that cells at lower passages are characterized by a peak at 1169 cm^−1^ that can be assigned to cholesterol esters.

### 2.5. NMR Exploratory Analysis of Cells

**Figure 7:**
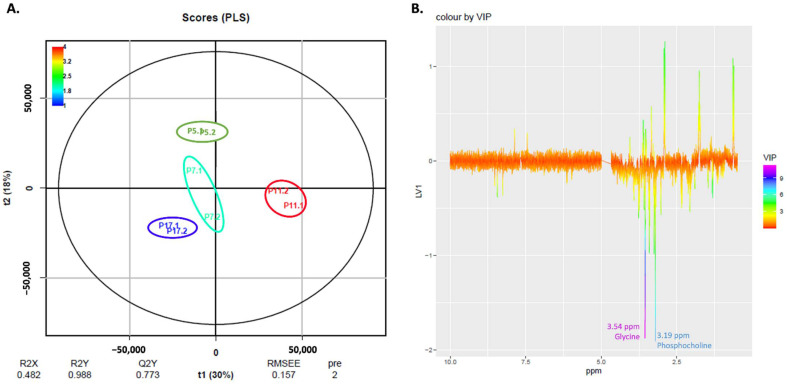
**PLS analysis of the aqueous endometabolome of human fibroblasts**. (**A**) Pareto PLS Scores Plot of the aqueous endometabolome of human fibroblasts. (**B**) Respective loadings plot showing VIP scores of factor 1.

To further study changes in the metabolome of cells during senescence, we performed an exploratory NMR analysis of the endo- and exometabolome of cells at passages 5, 7, 11 and 17, using two biological replicates for each passage. Representative NMR spectra of both endo- and exometabolome can be found in Figure S2A–C. To elucidate differences in cell metabolome during senescence, we conducted Pareto normalized PLS analyses on spectra of the endo- (both aqueous and organic fractions) and exometabolome. Figure 7A shows a PLS scores plot for the results from the aqueous endometabolome. In Figure 7B, the respective loadings plot colored with VIP scores is shown. It can be seen that factor 1 discriminates cells at passage 11 (positive t1) from the other passages (negative t1).

The most relevant VIP scores for this model are in purple and blue in the loadings plot (Figure 7B) and correspond to peaks at 3.19 and 3.54 ppm assigned to phosphocholine and glycine, respectively. These metabolites are increased in cells at passage 5, 7 and 17 compared to passage 11.

**Figure 8:**
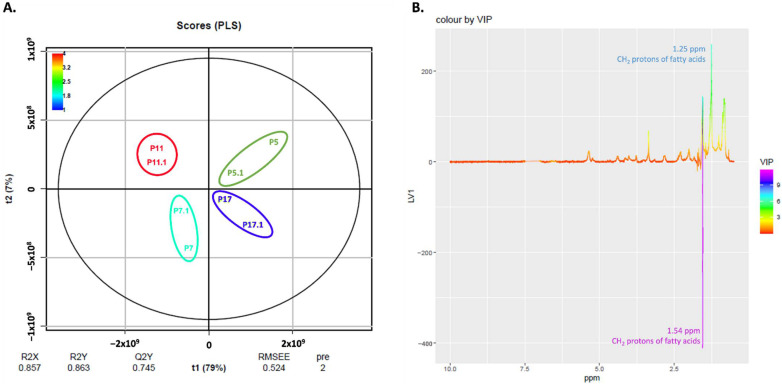
PLS **analysis of the lipidic endometabolome of human fibroblasts**. (**A**) Pareto PLS Scores Plot of the lipidic endometabolome of human fibroblasts. (**B**) Respective loadings plot showing VIP scores of factor 1.

The PLS analysis of the lipidic fraction from the endometabolome is shown in Figure 8. As for the aqueous fraction, t1 discriminates cells at passages 11 and 7 from cells at passages 5 and 17 (Figure 8A).

Looking at the loadings colored by VIP, cells at passages 11 and 7 are discriminated by a peak at 1.54 ppm. The cells at passages 5 and 17 are discriminated by a peak at 1.25 ppm (Figure 8B). These two peaks can be assigned to CH_2_ protons in the acyl chains of fatty acids.

**Figure 9:**
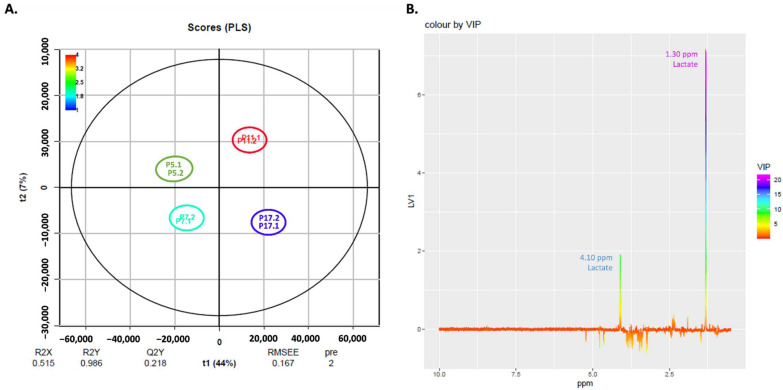
**PLS analysis of the exometabolome of human fibroblasts**. (**A**) Pareto PLS Scores Plot of the exometabolome of human fibroblasts. (**B**) Respective loadings plot showing VIP scores of factor 1.

The multivariate PLS analysis of the exometabolome, shown in Figure 9A, indicates that a positive t1 characterizes cells at higher passages (P11 and P17), and a negative t1 represents cells at initial passages (P5 and P7). The loadings, shown in Figure 9B, indicate that cells at passages 11 and 17 have higher amounts of lactate (peaks at 1.30 and 4.10 ppm).

To integrate all results and facilitate its comprehension, Table 1 summarizes the main changes detected by FTIR and NMR spectroscopy during the process of cell senescence in human fibroblasts.

## 3. Discussion

This study was designed to evaluate senescence-related changes in the spectroscopic profile of human fibroblasts, propose some spectroscopic markers of aged cells and, ultimately, extrapolate some understanding of the metabolic changes associated to physiological aging.

Cell culture studies continue to be widely used and have been increasing in the last years, in several research areas, including metabolomics [16]. They have great advantages compared to other disease models, namely, their reproducibility and control of the study environment. Among the various types of cells, primary cultures of human fibroblasts, namely, dermal fibroblasts, have been extensively used as models of diseases [19,20]. Similar to all somatic cells, fibroblasts enter replicative senescence [21]. From all cell lines, skin fibroblasts are long-lived cells that constantly suffer damage and adaptations, thus constituting a powerful indicator system of human aging [22,23]. Despite in vivo and in vitro aging present several differences, long-term cultures of human diploid cells, as fibroblasts, can provide valuable insights on the loss of replicative potential and senescence, an important mark of the aging process [14,15]. For instance, Porter and colleagues successfully used long-term cultures of neuronal cells to study aging in the brain [24].

Subculturing cells to study aging is not a new approach. In 1998, Benazzoug et al. subcultured dermal fibroblasts from passage 4 to passage 17 to study the effect of high glucose concentrations on the expression of collagen and fibronectin during cell aging [25]. On another study, Raffetto et al. subcultured fibroblasts from passage 1 to 6 and evaluate senescence by assessing senescence-associated β-gal activity [26]. In this study, human fibroblasts from a newborn were sub-cultured from passage 4 to passage 17, where standard biochemical methods indicate that a senescent phenotype (increased cell size, increase in the number of multinucleated cells (data not shown), increase in population doubling time (PDT) (Figure S3) and decreased levels of nuclear lamin B1 (Figure 1) is presented. In fact, lamin B1, a nuclear envelope protein involved in the regulation of nuclear stability, chromatin structure and gene expression, is considered to be a senescence marker and its levels significantly decrease in senescent cells [18]. Our Immunocytochemistry (ICC) results confirmed that a senescent process occurs in cultured fibroblast cells with an increasing number of passages, especially when passages 5 and 7 are compared to passages 11 and 17 (Figure 1). Moreover, we calculated population doubling time of cells (Figure S3), a common marker of aging in cell cultures [27,28] and results showed that cells at passage 17 more than tripled PDT comparing to initial passages (from 29.5 h in passage 5 to 101.2 h in passage 17), corroborating ICC results and morphological changes that show cells reached a senescent status.

FTIR spectroscopy is a widely used tool to exploratory metabolomic studies from cancer to aging and has been successfully used in cells [29,30,31,32]. Our FTIR data and PLS results revealed a strong correlation between spectroscopic profile and the cell passage number, indicating that senescence and ageing is affecting the metabolome and can be detected by FTIR.

Table 1 presents a summary of the metabolic alterations detected in cells during the process of cell senescence. For FTIR in the 3000–2800 cm^−1^ spectral region, results indicate that CH_2_ groups increase in younger fibroblasts but we see peaks associated with CH_3_ groups (2956 cm^−1^ and 2871 cm^−1^) in both early and late passage cells, respectively. In this case it is difficult to infer on acyl chain length by looking only to PLS-R analysis, despite we may infer that the increase in CH_2_ groups found in early passage cells may be indicative of longer lipid chains. In addition, the increase in the amount of CH groups in old fibroblasts may indicate an increase in unsaturation levels with age (Figure 4). When each passage was analyzed individually (using peak intensities), we confirmed that a linear decreasing tendency is observed up to passage 11, then an increasing tendency is observed for passage 17 (Figure 3A–C). On reaching senescence (passage 17) the fibroblasts return to peak intensity values similar to those seen at passage 5. This reflects spectroscopic profiles shown in Figure 4B, where spectroscopic profile of cells at passage 5 is similar to cells at passage 17. We may hypothesize that after a gradual decrease over time, later passage cells may present a lipid profile similar to what was already observed in long-lived people [33]. Nevertheless, looking to the correlation values of PLS-R analysis we observe that, rather than focus on values of a specific peak, looking to the overall changes in the spectroscopic profile over time may produce a more complete vision of the complex process that is cell senescence. For triglycerides, specifically the peak at 1741 cm^−1^, the results from peak intensities seem to indicate that there is a decrease in triglycerides as cells get older, except for cells at passage 17 (Figure 3). This is also shown by the PLS analysis, where the peak associated with triglycerides, is correlated with the oldest cells (Figure 5). FTIR spectroscopy also provides valuable information about the levels of cholesterol, in the 1200–900 cm^−1^ spectral region. Analysis of the intensity of the peak at 1169 cm^−1^, assigned to cholesterol esters, revealed a significant decrease from passage 5 to passage 11 followed by an increase to passage 17, still to levels significantly lower than the those seen for early passage cells (Figure 3). This is also corroborated by the PLS analysis, where this peak was associated with younger cells (Figure 6). The importance of studying the lipidome of aging has been reviewed by our group [34] and it has been found that lipids are so important for an organism that long-lived species undergo high selective pressure for genes involved in lipid synthesis and metabolism [35]. Our results here reveal that senescent cells present shorter lipid chains and in fact, a decrease in long-chain triglycerides has already been proposed as a marker of longevity [33,36]. Regarding lipid unsaturation, high levels of unsaturation were already reported to be related to increased lipid oxidation, which causes oxidative damage to cells, a feature of aging [37]. Other studies also report increased levels of unsaturated lipids in the elderly [38,39]. A previous fibroblast study by Gey and Seeger using NMR spectroscopy to evaluate cell senescence, found that senescent cells present higher levels of glycerophosphocholine [40].

FTIR results for the 1800–1500 cm^−1^ region provide important insights into changes in protein secondary structure. In fact, FTIR spectroscopy is widely used in the study of protein secondary structure in aqueous samples [41]. It is consensual that aging is accompanied by a decline in proteostasis, which may cause an increase in the levels of toxic proteins aggregates [42]. However, before analyzing protein secondary structure in detail it is important to point out that despite that fact that β-sheet-containing proteins can be considered aggregation-prone, there are proteins with high β-sheet content that do not usually aggregate [43]. In this study, total protein content (as determined by FTIR) did not follow a linear trend reflecting the complexity of protein nonlinear changes in metabolism upon age. During aging, the proteostasis network, which is highly dynamic, is responsible to keep the balance between protein synthesis and degradation, which, depending on the levels of cellular stress at a given timepoint, results in differences in the total protein levels. Our PLS results indicate that early passage cells are characterized by antiparallel β-sheets and by a peak at 1682 cm^−1^, associated with β-sheets and by using the ratio of the intensity of peaks associated with antiparallel β-sheets and total β-sheets we could verify that there is a progressive and significant increase in this ratio throughout the cell passages. Antiparallel β-sheets are present as small oligomers that are prone to aggregation [44]; therefore, the increase in the levels of antiparallel β-sheets during the process of cell senescence may indicate that cells at later passages present higher levels of toxic oligomers. In fact, an age-related accumulation of toxic oligomers in the brain has been seen [45]. Moreover, it is known that, in neurons, cell senescence can be triggered by the accumulation of toxic oligomers [46,47].

For intermolecular β-sheets, the PLS results did not reveal any definitive information; however, a peak area analysis showed a slight increase from passages 5 to 11 and a significant decrease between passage 11 and passage 17. These intermolecular β-sheet variations approaching senescence are corroborated by the amount of fibrillar structures in proteins we observed an increase from passage 5 to passage 11 but a drastic decrease at passage 17. These findings reflect changes in the secondary structure of existing proteins during aging and may reflect the loss protein homeostasis in the cells. The disruption of proteostasis is described as one of the hallmarks of aging [1]. The loss of function of the protective mechanisms against misfolded proteins, such as the ubiquitin-proteasome system or chaperone-mediated autophagy, results in an age-dependent accumulation of protein aggregates that is at the root of age-related diseases. In this work, the increase in the levels of intermolecular β-sheets and fibrillar structures (Figure 3) may reflect a progressive decline in the proteostasis machinery over the course of cell lifespan, until senescence. However, a significant decrease in fibrils and intermolecular β-sheets from passage 11 to passage 17 was noted when analyzing isolated peaks’ intensities. The senescence-associated secretory phenotype, may contribute to some age-related diseases [48]; however, senescence is a protective mechanism. The arrest of cell cycle can protect the organism from, for instance, malignant uncontrolled growth [48,49]. In this way, the pronounced decrease in the levels of fibrils from passage 11 to passage 17 may also be a marker of senescence. During aging (from passages 5 to 11), cells loss their ability to maintain proteostasis, which is reflected in the increasing levels of fibrillar structures (Figure 3). However, as cells reach a senescent status and stop dividing and growing, total protein levels decrease, also leading to a decrease in the levels of β-sheet structures. Another possible explanation for our results may be that cells at later passages produce proteins with lower content of β-sheet structures or even that senescent cell have higher levels of small toxic oligomers instead of bigger insoluble fibrillar structures, as proved by a progressive increase in these oligomeric structures over time.

FTIR spectroscopy in the 1200–900 cm^−1^ region can be used as a fingerprint of the sample. In fact, the high correlation found between spectral data and cell passage number means that FTIR spectroscopy can accurately predict cell passage number by looking at this region.

NMR results from the exometabolome revealed that lactate is associated with cells at passage 17 (Figure 9). Lactate is an endpoint of glycolysis and has an immunomodulatory effect (reviewed in [50]) and it has been proposed to act as a hormone, associated with neuroprotection in the brain [51]. Our NMR results for the endometabolome revealed that cells at later passages (P17) seem to present higher levels of intracellular phosphocholine and glycine (Figure 7) and, in fact, there are already some studies that associate phosphocholines with longevity [52].

This exploratory NMR analysis gave some insights into changes in metabolomic profile occurring during the process of cell senescence and aging. Despite the fact that we were only able to obtain two replicates at each timepoint, our results suggest that NMR may be used to follow metabolomic changes related to cell senescence and aging. However, further studies will need to be carried out using a higher number of replicates, to evaluate which intra- and extracellular metabolic pathways suffer the most pronounced changes during this process.

We understand that our results may raise some questions, in particular when comparing PLS analysis to specific peak intensities’ analysis: how can cells seem so different in the different passages (PLS analysis—Supplementary Figure S3) and then the results from peak intensity analysis show similar results for “younger” and “older” cells? Here, it is important to understand the added value of multivariate analysis in this type of work, when comparing to univariate analysis. As senescence is a complex process, that induces deep changes in cellular phenotype, measure just one or two molecules and compare its levels over time can be reductive because it does not give an overall vision of what is happen in the whole cell. Our results here specifically show that. If we look to specific peaks, associated to some structures, such as intermolecular β-sheets or even unsaturations, we see, in fact, that cells at later passage (P17) present levels of some structures that are similar to younger cells. However, using multivariate analysis, looking to the overall spectroscopic profile, we can see an overview of the process, because we are analyzing hundreds of variables at the same time, and our results clearly show that there is a good positive linear correlation between the cell passage and the spectroscopic profile. Therefore, looking to the spectroscopic profile of cells (in every analyzed regions), instead of looking to isolate molecules, allows us to predict if cells are near senescence or not.

In conclusion, our data suggest that, during the process of fibroblast senescence, cells undergo changes in their metabolic profiles. More important, the results show that to evaluate a process as complex as cell senescence, looking to the overall spectroscopic profiles over time, instead of focusing on specific molecules, may provide a complete overview of the process and can distinguish younger cells from those that are reaching senescence. The results for protein secondary structure suggest a progressive increase in fibrillar structures, followed by a decrease after senescence is reached. Later passage cells also seem to present higher levels of phosphocholine and glycine and extracellular lactate. Our results also suggest that FTIR spectroscopy, coupled with more sensitive approaches, such as NMR spectroscopy, can be successfully used to monitor the specific spectral signature of fibroblast senescence and give insights into the aging process. Since it is also possible to couple FTIR spectroscopy to fiber-optic probes, in the future, this methodology can be potentially used to easily assess the aging status of a tissue, for instance skin, in vivo, in a non-invasive way, and contribute to preventive medicine.

## 4. Materials and Methods

### 4.1. Cell Culture Procedures

The human fibroblast cell type AG22153, collected from the foreskin of a 1-day-old male (NIA Aging Cell Culture Repository, Apparently Healthy Collection, Coriell Institute for Medical Research, Camden, NJ, USA) was cultured in a 1:1 mixture of Dulbecco’s Modified Eagle’s Medium (DMEM) and Ham’s F-12 Nutrient Mixture (Gibco, Thermo Fisher Scientific, Waltham, MA, USA) supplemented with 10% fetal bovine serum (FBS; Gibco, Thermo Fisher Scientific, Waltham, MA, USA). Cells were maintained at 37 °C with 5% CO_2_ in a humidified chamber and sub-cultured at 80–90% confluency. Culture media was replaced every two days. Cells were sub-cultured from passage 3 to passage 17.

### 4.2. Immunocytochemistry (ICC)

Human Fibroblasts at passages 5, 7, 11 and 17 were fixed with a 4% paraformaldehyde (PFA) solution for 20 min. Posteriorly, cells were incubated for 10 min at room temperature (RT) with a permeabilization solution (0.2% Triton X-100 in PBS 1×). Following blocking with 5% BSA in PBS for 2 h at RT, cells were incubated with the primary antibody against Lamin B1 (sc-56144, Santa Cruz Biotechnology, TX, USA), diluted in 5% BSA in PBS 1×, for 2 h at RT, followed by incubation with Alexa Fluor 488-conjugated secondary antibody (A-11001, Invitrogen, MA, USA), diluted in 5% BSA in PBS 1×, for 1 h at RT in the dark. Coverslips were mounted on microscope slides using Vectashield anti-fade mounting medium with DAPI (Vector Laboratories, Burlingame, CA, USA) and subsequently analyzed by Confocal microscopy.

### 4.3. Confocal Microscopy

ICC preparations were visualized under a Zeiss LSM 880 confocal laser scanning microscope with an Airyscan (Zeiss, Jena, Germany) and a 63× oil immersion objective. Laser lines Diode 405 nm and Argon 488 nm were used to excitation of fluorescence of DAPI and Alexa Fluor 488, respectively. Z-stacks were produced by acquiring microphotographs in multiple optical sections of the Z-axis. Corrected total cell fluorescence (CTCF) of Lamin B1 in fibroblasts’ nuclei was quantified using ImageJ software [53,54]. The sum of the intensities of the z-stacks was used in ImageJ to create a Z projection and then nuclei were manually detected as regions of interest using the DAPI channel. CTCF was calculated for each nucleus by subtraction of the fluorescence of adjacent background. For each experiment, a total of 30 nuclei were counted and CTCF was calculated, as previously described [55]. For each cell passage, three experiments were made.

### 4.4. Fourier-Transform InfraRed (FTIR) Spectroscopy

#### 4.4.1. Cell Pellet Preparation

When reaching 80–90% of confluency, cells were detached from culture flasks with trypsin, using a standard protocol for subculturing cells [56] and an aliquot of the cell suspension was used for counting the number of viable cells in a hemocytometer, with Trypan Blue, as previously described [57]. Then, two aliquots containing 500,000 cells each were centrifuged for 3 min, 3000× *g* at RT. After that, culture media was discarded and cells were washed with PBS and centrifuged again (3 min, 3000× *g*, RT). PBS was removed and cells were immediately frozen at −80 °C until FTIR analysis. The number of cells to use was optimized before starting experiments and 500,000 cells were enough to produce high-quality spectra.

#### 4.4.2. FTIR Spectra Measurements and Spectra Preprocessing

FTIR spectra of cells from passages 4 to 17 were obtained in mid-infrared range (4000–600 cm^−1^) using an ATR-FTIR Bruker Alpha Platinum spectrometer (Bruker^©^, Billerica, MA, USA), coupled to OPUS software (Bruker^©^, Billerica, MA, USA) [58]. The resolution used was 8 cm^−1^, with 64 scans co-added. All FTIR spectra acquisition was performed in a room with controlled temperature and relative humidity (23 °C and 35%, respectively). The cell pellet was placed at the center of ATR and air dried. The drying process was monitored by visual observation of the live spectrum at OPUS software and the sample was considered dried when spectrum profile did not change. For each passage, five biological replicates and three technical replicates were acquired. Between each sample, a background spectrum was acquired against air, and the ATR crystal was cleaned with 70% ethanol and distilled water.

FTIR spectra were exported in OPUS format and imported to The Unscrambler X software (V.10.5., Camo Analytics, Oslo, Norway) [59]. All spectra were individually visually analyzed and spectra with background noise or with suspicious profile were repeated to ensure good quality data. After visual inspection, a PCA analysis was performed to identify and remove outliers, as previously described (reviewed in [60]). Samples with high values for PCA Q-residuals were considered outliers and removed from data matrix.

Spectra were divided into three main spectral regions: 3050–2800 cm^−1^, 1800–1500 cm^−1^ and 1200–900 cm^−1^, baseline-corrected and area-normalized. Normalized spectra were then derived using 2^nd^ derivative with the Savitzky–Golay algorithm and 3 smoothing points. The 2nd derivatives were used to deconvolute bands to extract more detailed information and reduce variability among replicates. Pre-processed spectra were then subjected to both multivariate analysis (PLS-R) and analysis of specific peak intensity.

#### 4.4.3. PLS-R Multivariate Analysis

With spectroscopic data, it is virtually impossible to analyze all variables without the use of multivariate analysis. Here, we used PLS-R supervised multivariate analysis in the three spectral regions, using the 2nd derivative spectra and a random intern cross-validation with Kernel algorithm, as previously explained [10]. In this case, the Y matrix is the cell passage number which was used to produce a correlation plot between spectral profile and passage number. PLS-R was built using 3 factors for all the three regions. Multivariate analysis was performed using The Unscrambler X software (v.10.5 CAMO Analytics, Oslo, Norway) [59].

#### 4.4.4. Analysis of Peak Intensities

The intensity of specific spectral bands from cells at passages 5, 7, 11 and 17 was calculated using two different approaches as in our previous study [10]. Briefly, the intensity of peaks assigned to CH (3010 cm^−1^), CH_2_ (2851 cm^−1^ and 2922 cm^−1^) CH_3_ (2959 cm^−1^ and 2871 cm^−1^), C=O (1733 cm^−1^), cholesterol esters (1169 cm^−1^) and protein secondary structures, namely β-sheets (1693 cm^−1^, 1682 cm^−1^ and 1628 cm^−1^), were calculated using the peak intensities of 2nd derivative spectra. For the calculation of the fibril formation ratio and total protein amount, we used non-derivative spectra to extract the values of the intensity of Amide I and Amide II peaks.

### 4.5. NMR Spectroscopy

#### 4.5.1. Quenching and Extraction of the Cell Extracts: Endo- and Exometabolome

NMR analysis was performed on cells at passages 5, 7, 11 and 17, using two biological replicates. For the extraction of the endometabolome, approximately 9 × 10^6^ cells were used. Cells were put on ice and a protocol for the chloroform-methanol extraction of metabolites by was used [61]. The 10 mL of culture media, from one confluent cell culture flask (approximately 9 × 10^6^ cells), was stored at 4 °C during endometabolome extraction. Briefly, cells were quickly incubated with methanol and scraped from the flask. Then, after chloroform extraction and centrifugation, the aqueous and lipophilic phases were carefully separated into a microtube and to an amber glass vial, respectively. The aqueous phase was dried under vacuum, in a Scanspeed MaxiVac (LaboGene, Lillerød, Denmark), for approximately 4 h. The lipophilic phase was left open in a fume hood until complete evaporation of the solvent (approximately 2 h). The extracts were then stored at 4 °C until further analysis. For exometabolome extraction, culture media was centrifuged at 1000× *g* for 5 min and then 1 mL of the supernatant was transferred to a microtube and frozen at −80 °C.

For the NMR analysis, aqueous extracts were re-suspended in 620 µL of a buffer composed by 100 mM Na_2_HPO_4_ in D_2_O, at pH 7.0, with 0.01% TSP. Lipidic extracts were re-suspended in CDCl_3_ with 0.3% TMS (*v/v*). All samples were then centrifuged for 1 min, at 16,000× *g* and RT. The ^1^H-NMR spectra were recorded at 298 K on a Bruker Avance III 500 MHz spectrometer (Bruker^©^, Billerica, MA, USA). The 1D proton spectra were acquired using a NOESY pulse sequence to suppress the water resonance, with a sweep width of 7002 Hz (14 ppm), 32 k data points, recycle delay 6 s, mixing time 100 ms and 128 scans per free induction decay.

#### 4.5.2. NMR Spectral Processing

Spectra were processed with zero filling to 64 k data points and 0.3 Hz line broadening using iNMR software (Nucleomatica, Molfetta, Italy) [62]. All spectra were manually phased, baseline-corrected and exported as matrices. These matrices were area normalized, overlaid and checked in iNMR and aligned and processed in RStudio (Boston, MA, USA) [63] using in-house packages and the published speaq [64] and ropls package [65]. The water (4.85–4.65 ppm) and chloroform (7.50–7.00 ppm) regions were excluded from the matrices.

#### 4.5.3. Multivariate Analysis

Pareto-PLS multivariate analysis was performed using the ropls package [65] in RStudio. The identification of metabolites was carried out by comparing spectra with those of standard compounds from the Human Metabolome Database and the Magnetic Resonance Data Bank. Lipid metabolites were identified using the assignments from [61].

### 4.6. Statistical Analysis

For ICC quantitative results and analysis of FTIR peak intensities, statistical analysis was performed using GraphPad Prism 6.0 software (GraphPad software, CA, USA) [66]. To compare levels of Lamin B1 or FTIR peak intensities between cell passages 5, 7, 11 and 17, the parametric Ordinary One-Way ANOVA test, followed by Tukey’s multiple comparison test, or the non-parametric Kruskal–Wallis test, followed by the Dunn’s multiple comparison test, were applied, depending on the normal or non-normal distribution of the data. The results were expressed as mean ± standard deviation and were considered statistically significant when *p* < 0.05.

## Figures and Tables

**Table 1:** Summary of FTIR and NMR results of young versus old human fibroblasts.

Young Fibroblasts	Old Fibroblasts
↑ CH_2_ groups of lipids	↑ CH_3_ groups of lipids (except for cells at passage 17)
	↑ CH groups of lipids
	↓ triglycerides (except for cells at passage 17)
	↓ cholesterol (except for cells at passage 17)
	↑ antiparallel β-sheet structures of proteins
	↑ intermolecular β-sheet structures of proteins (except for cells at passage 17)
	↑ phosphocholine
	↑ glycine
	↑ extracellular lactate

↑ increase; ↓ decrease.

## Data Availability

Not applicable.

## Supplementary Materials

The following supporting information can be downloaded at: https://www.mdpi.com/article/10.3390/ijms23105830/s1.
