# Supplementary material for: The Long-Term Culture of Human Fibroblasts Reveals a Spectroscopic Signature of Senescence
Source: Int J Mol Sci. 2022 May 23;23(10):5830. doi: 10.3390/ijms23105830 (PMC9146002; doi:10.3390/ijms23105830)
Supplement: Supplementary file 1 [file ijms-23-05830-s001.zip › ijms-1654073-supplementary.pdf]

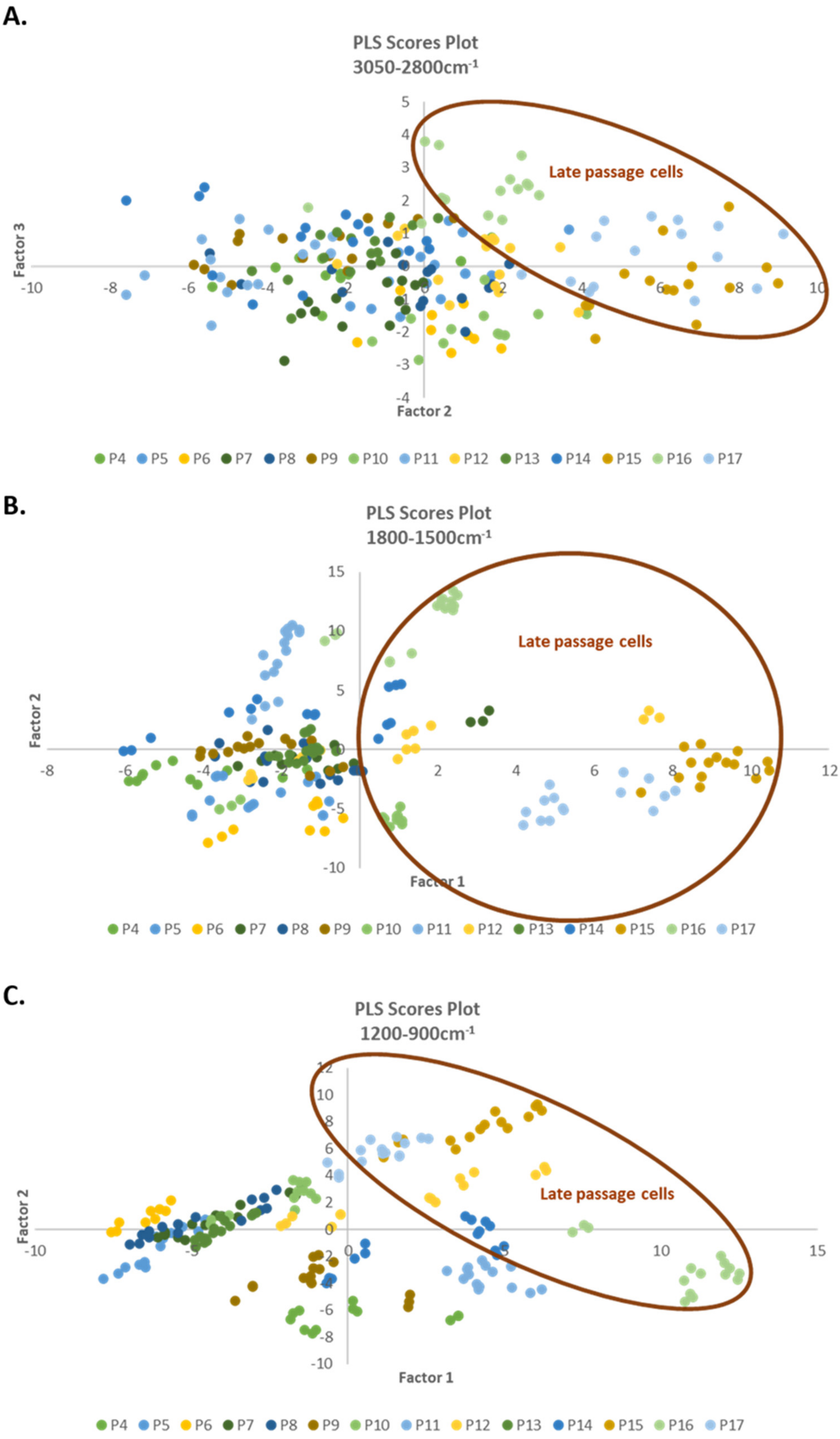

Figure S1: PLS Scores Plot for cells from passages 4 to 17 in the A. 3050-2800cm^{-1}, B. 1800-1500cm^{-1} and C. 1200-900cm^{-1} spectral regions.

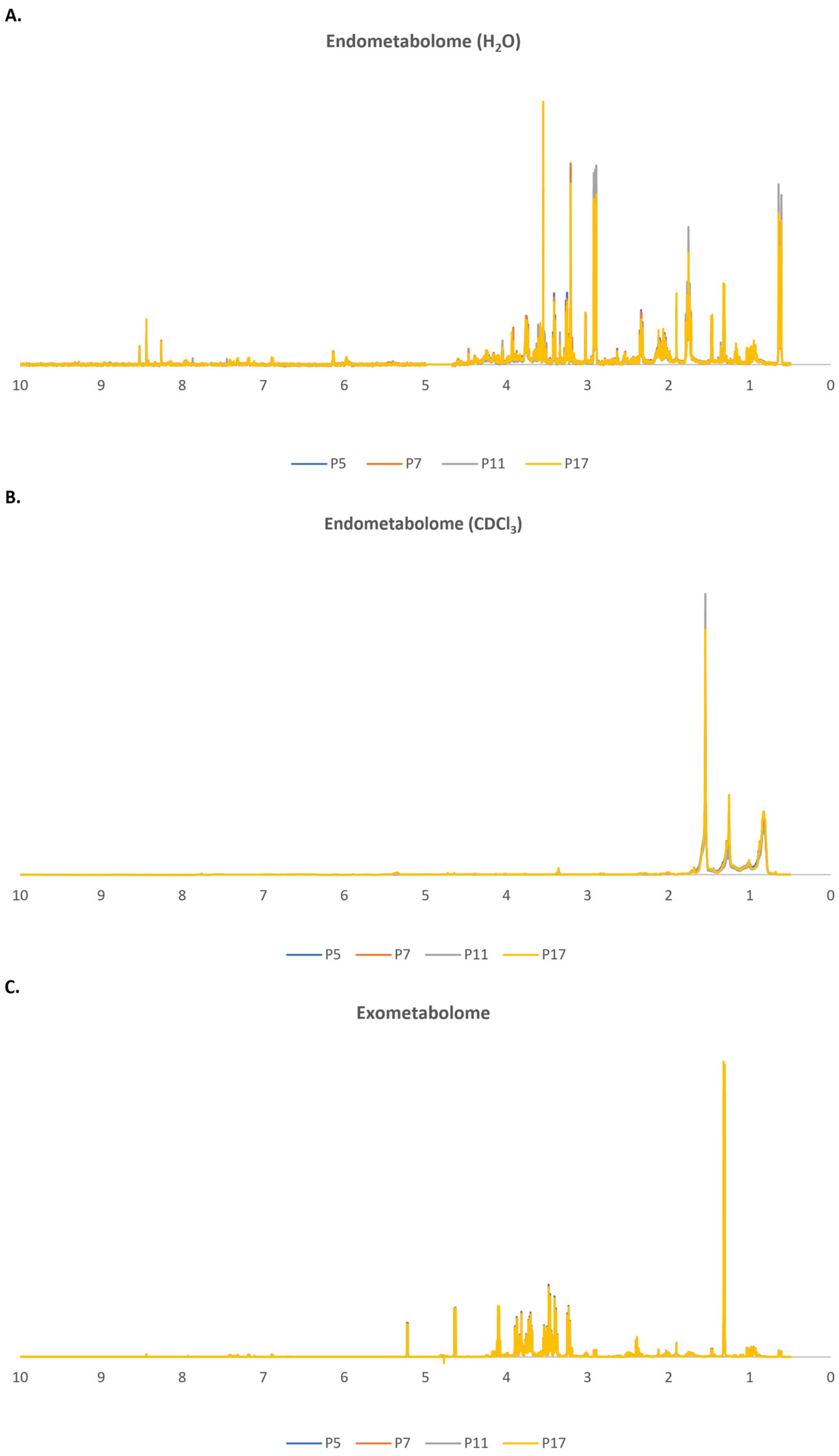

Figure S2: Representative NMR spectra of aqueous endometabolome (A), lipidic endometabolome (B) and exometabolome (C) of dermal fibroblasts at passages 5, 7, 11 and 17.

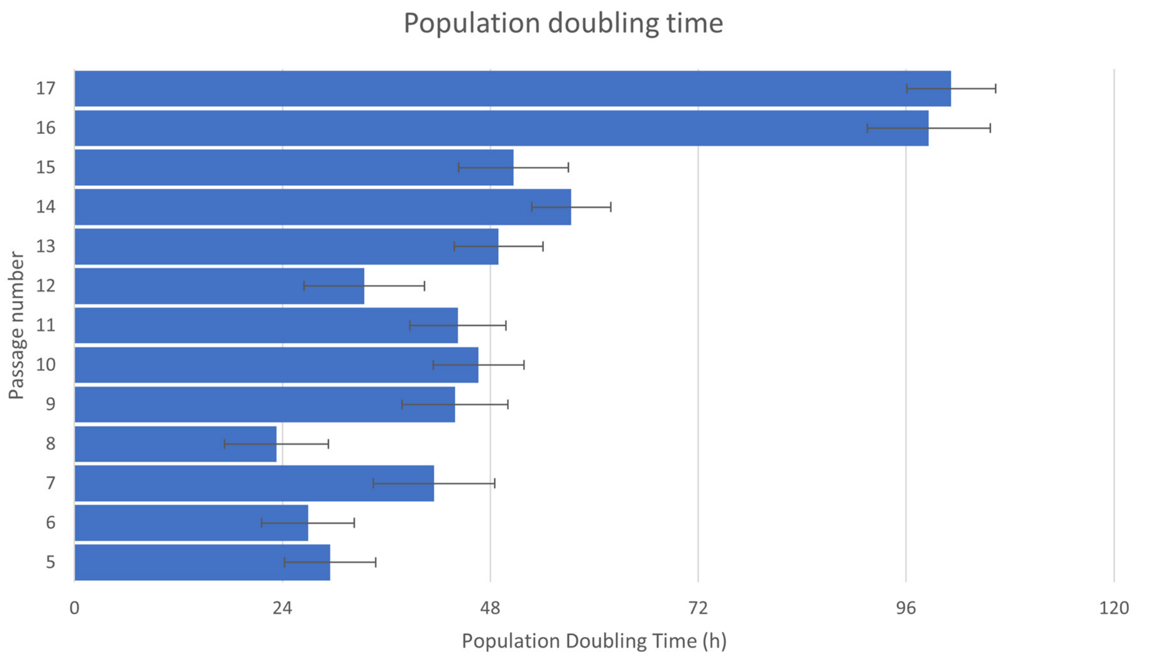

Figure S3: Population doubling time of human dermal fibroblasts from passages 5 to 17 (AG22153, NIA Aging Cell Culture Repository, Apparently Healthy Collection, Coriell Institute for Medical Research, New Jersey, USA). Population doubling time was calculated using the formula: PDT = D*(ln(2)/ln(CF/CI)), according to the literature [31]. Data is presented as mean ± standard deviation. PDT = population doubling time; D = duration of cell culture, in hours; CF = final concentration of cells (cells/mL); CI = initial concentration of cells (cells/mL).
